# *Spirosoma* *endophyticum* sp. nov., isolated from Zn- and Cd-accumulating *Salix caprea*

**DOI:** 10.1099/ijs.0.052654-0

**Published:** 2013-12

**Authors:** Julia Fries, Stefan Pfeiffer, Melanie Kuffner, Angela Sessitsch

**Affiliations:** AIT Austrian Institute of Technology GmbH, Bioresources Unit, Tulln, Austria

## Abstract

A Gram-reaction-negative, yellow-pigmented strain, designated EX36^T^, was characterized using a polyphasic approach comprising phylogenetic, morphological and genotypic analyses. The endophytic strain was isolated from Zn/Cd-accumulating *Salix caprea* in Arnoldstein, Austria. Analysis of the 16S rRNA gene demonstrated that the novel strain is most closely related to members of the genus *Spirosoma* (95 % sequence similarity with *Spirosoma linguale*). The genomic DNA G+C content was 47.2 mol%. The predominant quinone was and the major cellular fatty acids were summed feature 3 (iso-C_15 : 0_ 2-OH and/or C_16 : 1_ω7*c*), C_16 : 1_ω5*c*, iso-C_17 : 0_ 3-OH and iso-C_15 : 0_. On the basis of its phenotypic and genotypic properties, strain EX36^T^ should be classified as a novel species of the genus *Spirosoma*, for which the name *Spirosoma* *endophyticum* sp. nov. is proposed. The type strain is EX36^T^ ( = DSM 26130^T^ = LMG 27272^T^).

The genus *Spirosoma* was first proposed by Larkin & Borrall (1984) and belongs to the family *Flexibacteraceae* in the phylum *Bacteroidetes*. At the time of writing the genus *Spirosoma* includes five species, the type species *Spirosoma linguale* (Larkin & Borrall, 1984), *Spirosoma rigui* (Baik *et al.*, 2007), *Spirosoma panaciterrae* (Ten *et al.*, 2009), *Spirosoma spitsbergense* and *Spirosoma luteum* (Finster *et al.*, 2009). So far, *Spirosoma* strains have been isolated from various habitats, such as fresh water, permafrost soil or soil from a ginseng field. Strain EX36^T^, which is proposed in this study to represent a novel species, was isolated in course of the analysis of bacteria associated with the heavy metal accumulating plant *Salix caprea* (Kuffner *et al.*, 2010).

For the isolation of strain EX36^T^, *Salix caprea* trees growing on a former Zn/Pb mining and processing site in Arnoldstein (Austria) were sampled (Kuffner *et al.*, 2010). Xylem sap extract was directly plated on 10 % tryptic soy agar (TSA, Merck Darmstadt, Germany) and after 1 week of incubation single colonies were picked and streaked on phosphate-poor MOPS medium (Neidhardt *et al.*, 1974) containing 0.1 % glucose and 1 mM ZnSO_4_. The strain was routinely cultured on 10 % TSA. For maintenance, the cell material was suspended in 10 % tryptic soy broth (TSB, Merck, Darmstadt, Germany) containing 15 % glycerol and stored at −80 °C. Endophytic colonization was confirmed by inoculating two maize and two potato cultivars, growing the plants under *in vitro* conditions and reisolating the strain from root and stem tissues.

For the extraction of bacterial DNA the Gen Elute Bacterial Genomic DNA kit (Sigma–Aldrich) was used. The 16S rRNA gene was amplified by PCR using the primers 8f (5′-AGAGTTTGATCCTGGCTCAG-3′) (Weisburg *et al.*, 1991) and 1520r (5′-AAGGAGGTGATCCAGCCGCA-3′) (Edwards *et al.*, 1989). Sequencing of the amplified PCR product was performed by LGC Genomics (Berlin, Germany). The obtained partial sequences were assembled using the programs BioEdit (Hall, 1999) and seqman pro (DNAstar). The consensus sequence was subjected to nucleotide blast analysis (http://blast.ncbi.nlm.nih.gov/Blast.cgi) to search the database of the National Center for Biotechnology Information (NCBI) for the closest relatives of the bacterial strains with validly published names. Sequence comparisons indicated that the isolate belonged to the family *Flexibacteraceae*.

Nearly complete 16S rRNA gene sequences of strain EX36^T^ and of all species of the genus *Spirosoma* with validly published names and of selected species of the family *Cytophagaceae*, which were downloaded from the NCBI GenBank sequence database, were imported into the arb program package (Ludwig *et al.*, 2004). Sequences were aligned into the silva SSURef 102 (Pruesse *et al.*, 2007) database by using the option ‘autosearch by PT_server’ of the arb editor. Alignments were manually corrected using the arb editor. A maximum-likelihood phylogenetic tree was reconstructed using RAxML v. 7.4.2 (Stamatakis, 2006a) by execution of the following command line in raxmlGUI v. 1.3 (Silvestro & Michalak, 2012): raxmlHPC.exe -T 2 <number of processors >-f a -m GTRGAMMA -x 336 <seed1 >-p 115 <seed2 >-N 100 <bootstraps >-o CarHomin <outgroup >-s <input file >-O <output order >. We used a combination of the Gamma model of rate heterogeneity (Yang, 1994) and the CAT model (Stamatakis, 2006b), which was implemented in the rapid bootstrapping algorithm, (Stamatakis *et al.*, 2008) was performed with 100 replicates and using general time reversible (GTR) as the substitution matrix. In Fig. 1 the position of EX36^T^ in the distinct cluster of the genus *Spirosoma* can be clearly recognized. The calculation of pairwise sequence similarity using a global alignment algorithm (Myers & Miller, 1988), which was implemented at the EzTaxon-e server (http://eztaxon-e.ezbiocloud.net/; Kim *et al.*, 2012) showed highest sequence similarity values for strain EX36^T^ to *Spirosoma linguale* DSM 74^T^ (95.7 %), followed by *S. luteum* SPM-10^T^ (93.9 %), *S. spitsbergense* SPM-9^T^ (93.9 %), *S. rigui* KCTC 12531^T^ (93.8 %) and *S. panaciterrae* Gsoil 1519^T^ (92.5 %).

**Fig. 1.**
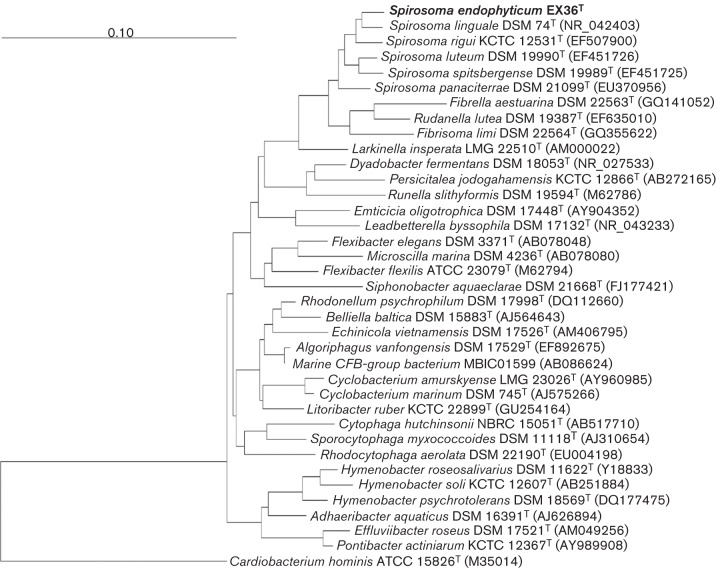
Maximum-likelihood tree (bootstrap: 100 replicates) based on 16S rRNA gene sequence data (sequence length 1296 bp) showing the phylogenetic position of strain EX36^T^ among related species selected from the phylum *Bacteroidetes*. *Cardiobacterium hominis* ATCC 15826^T^ (M35014) was used as an outgroup.

Growth of strain EX36^T^ was tested at various temperatures (4, 20, 23, 28, 37 and 41 °C) on 10 % TSA plates for up to 1 week. The pH range for growth (pH 4, 5, 6, 7, 8 and 9) was determined by measuring OD_600_ changes in cultures incubated at 28 °C with shaking at 190 r.p.m. compared with an uninoculated control. Salt tolerance was determined by amending 10 % TSB with NaCl to final concentrations of 0.1, 0.2, 0.3, 0.4, 0.5, 0.6, 0.8, 1.0, 2.0, 3.0, 4.0, 5.0 and 10.0 % NaCl (w/v). The Gram reaction of strain EX36^T^ was determined by using the non-staining method described by Buck (1982). Pigment analysis of cells grown on 10 % TSA was performed in triplicates by extraction with acetone according to the method described by Denner *et al.* (2001) using a U-2900 spectrophotometer (Hitachi). Minimal inhibition concentrations (MIC) for Zn and Cd were determined according to the method of Kuffner *et al.* (2008). Additionally cells were tested for flexirubin pigments using the method described by Bernardet *et al.* (2002). Oxidase and catalase activity were tested as outlined by Smibert & Krieg (1994). Additional biochemical tests were performed by the Identification Service of the DSMZ (Leibniz-Institut DSMZ-Deutsche Sammlung von Mikroorganismen und Zellkulturen GmbH, Braunschweig, Germany) using API 20NE (bioMérieux) and GENIII plates (Biolog). Cell morphology after 4 days of growth at 28 °C was investigated using fluorescence and bright-field microscopy (IX81, Olympus; Axiovert 200 M, Zeiss). Antibiotic susceptibility was determined by the disc diffusion method on 10 % TSA plates.

Cells of strain EX36^T^ were rod-shaped, Gram-reaction-negative and 1.2×2−17.5 µm in size (Fig S1, available in IJSEM Online). Most cells were arranged in pairs, but filaments up to 55 µm were observed. EX36^T^ showed yellowish, opaque, semi-translucent colonies with a smooth and shiny surface and a circular and convex shape. The diameter of colonies grown on 10 % TSA at 28 °C for 1 week varied between 1.5 and 3.0 mm. The strain was positive for catalase and oxidase activity; detailed results of biochemical and physiological analyses are listed in Table 1 and in the species description. In contrast to other species of the genus *Spirosoma*, cells of EX36^T^ showed a length up to 17.5 µm, did not grow at 5 and 42 °C, did not tolerate NaCl concentrations higher than 0.6 % (w/v), had the lowest genomic G+C content and showed differences in antibiotic susceptibility. Low tolerance of Cd and Zn was observed (slow growth at 4 mM Zn and 1 mM Cd). The analysis of yellow pigments showed three absorption maxima at 428, 453 and 483 nm. EX36^T^ was negative for flexirubin-type pigments.

**Table 1.** Differential characteristics of strain EX36^T^ and recognized species of the genus *Spirosoma* Strains: 1, EX36^T^ (data from this study); 2, *S. linguale* DSM 74^T^ (Larkin & Borrall, 1984; and this study); 3, *S. luteum* DSM 19990^T^ (Finster *et al.*, 2009); 4, *S. spitsbergense* DSM 19989^T^ (Finster *et al.*, 2009); 5, *S. rigui* KCTC 12531^T^ (Baik *et al.*, 2007); 6, *S. panaciterrae* DSM 21099^T^ (Ten *et al.*, 2009). All strains are catalase-positive, Gram-reaction-negative and negative for nitrate reduction, utilization of gluconate, caprate, adipate and glycerol. +, Positive; −, negative; w, weakly positive; nd, not determined; r, resistant; s, susceptible.

Characteristic	1	2	3	4	5	6
Cell morphology	Rod-shaped, filaments up to 55 µm	Horseshoe-shaped, coils, spirals, filaments	Rod-shaped, filaments up to 80 µm	Rod-shaped, filaments up to 80 µm	Rod-shaped, filaments up to 54 µm	Rod-shaped
Cell size (µm)	1.2×2−17.5	1×6	1×4−10	1×4−10	0.8−1.0×1.5−10.8	nd
Growth at:						
42 °C	−	−	−	−	−	+
5 °C	−	+	+	+	+	−
Optimum temperature (°C)	28	20−30	25	25	30	30
NaCl range for growth (%, w/v)	0−0.6	0−1.25	0−1	0−1	0−1	0−1
Assimilation of:						
Cellobiose	w	+	−	−	−	+
d-Fructose	w	+	−	−	−	−
d-Galactose	−	+	nd	nd	−	−
Maltose	w	+	−	−	−	+
Melibiose	w	+	nd	nd	−	+
Raffinose	+	+	nd	nd	−	−
l-Rhamnose	−	+	nd	nd	−	−
Enzymic activities						
Aesculin hydrolysis	+	nd	+	−	+	nd
Arginine dihydrolase	−	+	−	−	nd	−
β-Galactosidase	w	+	−	−	+	−
Oxidase	+	+	−	w	−	+
Urease	−	−	−	+	nd	−
Antibiotic Susceptibility						
Chloramphenicol	s	r	r	s	s	nd
Kanamycin	s	s	s	s	r	nd
DNA G+C content (mol%)	47.2	51−53	50.2	49.1	53.3	50.1

Analyses of cellular fatty acid composition, respiratory quinones, polar lipids and chromosomal G+C content were performed by the Identification Service of the DSMZ. The fatty acid profile was determined according to the protocol of the Microbial Identification System (MIDI). The major fatty acids of strain EX36^T^ were summed feature 3 (iso-C_15 : 0_ 2-OH and/or C_16 : 1_ω7*c*; 49.3 %), C_16 : 1_ω5*c* (23.8 %), iso-C_17 : 0_ 3-OH (6.2 %) and iso-C_15 : 0_ (5.4 %). A detailed overview of the cellular fatty acid profiles of all species of the genus *Spirosoma* can be found in Table 2. Differences between the fatty acid profile of EX36^T^ and other species of the genus *Spirosoma* were found in the amounts of iso-C_15 : 0_, C_16 : 1_ω5*c* and summed feature 3. In contrast to *S. linguale* DSM 74^T^, the fatty acids C_15 : 0_ and anteiso-C_15 : 0_ were not detected.

**Table 2.** Fatty acid profiles (%) of strain EX36^T^ and its closest phylogenetic neighbours from the genus *Spirosoma* Strains: 1, EX36^T^ (data from this study); 2, *S. linguale* DSM 74^T^ (data from this study); 3, *S. luteum* DSM 19990^T^ (Finster *et al.*, 2009); 4, *S. spitsbergense* DSM 19989^T^ (Finster *et al.*, 2009); 5, *S. rigui* KCTC 12531^T^ (Baik *et al.*, 2007); 6, *S. panaciterrae* DSM 21099^T^ (Ten *et al.*, 2009). tr, Trace amount (<1 %); −, not detected.

Fatty acid	1	2	3	4	5	6
Straight chain saturated						
C_14 : 0_	tr	tr	tr	tr	1.3	1.6
C_15 : 0_	−	2.2	tr	tr	−	2.7
C_16 : 0_	3.9	3.9	7.3	7.0	8.8	5.2
C_16 : 0_ 3-OH	2.4	2.3	3.5	4.5	2.8	−
C_18 : 0_	−	−	−	−	1.2	−
Branched saturated						
iso-C_13 : 0_	2.3	2.3	−	−	−	−
iso-C_15 : 0_	5.4	9.5	2.6	5.7	9.5	15.1
iso-C_15 : 0_ 3-OH	3.2	3.0	2.5	2.3	2.6	−
iso-C_16 : 0_ 3-OH	−	−	−	tr	−	−
iso-C_17 : 0_ 3-OH	6.2	6.0	4.7	6.1	3.5	3.2
anteiso-C_15 : 0_	−	2.2	−	−	1.3	1.9
anteiso-C_15 : 0_ 3-OH	−	−	tr	1.1	−	−
Mono-unsaturated						
C_16 : 1_ω5*c*	23.8	22.5	19.1	21.3	18.5	33.4
C_18 : 1_ω7*c*	−	−	−	−	1.5	−
Summed feature 3*	49.3	41.1	45.0	42.9	45.6	35.5

* Summed feature 3 represents groups of two or three fatty acids that could not be separated by GLC with the MIDI system; summed feature 3 contained iso-C_15 : 0_ 2-OH and/or C_16 : 1_ω7*c*.

The predominant menaquinone, in accordance with all other species of the genus *Spirosoma*, was MK-7. As polar lipids, phosphatidylethanolamine, two aminophospholipids, two aminolipids, a glycolipid and three unknown lipids were detected on the TLC plate. The DNA G+C content of strain EX36^T^ was 47.2 mol%, which is lower than reported values for all other species of the genus *Spirosoma* with validly published names.

The analysis of DNA−DNA similarity of strain EX36^T^ with its nearest phylogenetic neighbour *S. linguale* DSM 74^T^ was also carried out by the Identification Service of the DSMZ. The experiment was performed in duplicates. DNA−DNA hybridization showed a DNA−DNA similarity of 12.2 % (second measurement: 17.2 %), demonstrating that these two strains do not represent the same species.

The present data regarding 16S rRNA gene sequence analysis, physiological, chemotaxonomic and morphological properties indicates, that strain EX36^T^ represents a distinct species in the genus *Spirosoma*, for which the name *Spirosoma* *endophyticum* sp. nov. is proposed.

## Description of *Spirosoma* *endophytica* sp. nov.

*Spirosom endophyticum* (en.do.phy′ti.cum. Gr. Pref. *endo* within; Gr. n. *phyton* plant; L. neut. suff. -*icum* adjectival suffix used with the sense of belonging to; N.L. neut. adj. *endophyticum* within plant, referring to the endophytic nature of the strain and its isolation from plant tissue).

Cells are rod-shaped, Gram-reaction-negative, non-spore-forming, with a size of 1.2×2–17.5 µm. A yellow pigment which is not of the flexirubin type is produced. Filaments up to 55 µm may be formed. Colonies on 10 % TSA are opaque, semi-translucent with a smooth and shiny surface and a circular, convex shape. Aerobic growth occurs at 20–28 °C (optimum at 28 °C), pH 5–8 (optimum at pH 7); tolerates concentrations up to 0.6 % NaCl (w/v) in the medium, whereas best growth was achieved in absence of NaCl. Positive for catalase and oxidase activity. Nitrate is not reduced and indole is not produced. Negative for glucose fermentation, hydrolysis of arginine and gelatin, and urease activities and positive for aesculin hydrolysis. Does not utilize the following substrates: arabinose, mannitol, *N*-acetylglucosamine, gluconate, caprate, adipate, malate, citrate, phenylacetate, β-methyl d-glucoside, d-salicin, n-acetyl-β-d-mannosamine, n-acetyl neuraminic acid, d-galactose, d-fucose, l-fucose, l-rhamnose, inosine, d-arabitol, *myo*-inositol, d-aspartic acid, d-serine, glycyl-l-proline, l-alanine, l-arginine, l-aspartic acid, l-glutamic acid, l-serine and pectin. The following substrates are weakly utilized: dextrin, maltose, trehalose, cellobiose, gentiobiose, sucrose, turanose, stachyose, α-lactose, melibiose, α-d-glucose, d-mannose, d-fructose, d-mannitol and l-histidine. d-Raffinose and *N*-acetyl-d-glucosamine are utilized. Susceptible to the following antibiotics (µg per disc): streptomycin (10), kanamycin (30), chloramphenicol (60) and rifampicin (15) and resistant to ampicillin (10), polymyxin B (20), tetracycline (15) and erythromycin (15). The major fatty acids are summed feature 3 (iso-C_15 : 0_ 2-OH and/or C_16 : 1_ω7*c*), C_16 : 1_ω5*c*, iso-C_17 : 0_ 3-OH and iso-C_15 : 0_; the complete fatty acid profile can be found in Table 2. The predominant menaquinone is MK-7. The major polar lipid is phosphatidylethanolamine.

The type strain, EX36^T^ ( = DSM 26130^T^ = LMG 27272^T^), was isolated from Zn/Cd-accumulating *Salix caprea* in Arnoldstein, Austria. The DNA G+C content of the type strain is 47.2 mol%.
